# Did Inequalities in Mothers’ and Children’s Health and Well-Being in Japan Increase through the Pandemic? Evidence from Nationwide Surveys and Routinely Collected Data

**DOI:** 10.3390/children11030330

**Published:** 2024-03-09

**Authors:** Hajime Takeuchi, Yoichi Satoh, Shanti Raman, Nick Spencer

**Affiliations:** 1 School of Social Welfare, Bukkyo University, Kyoto 603-8301, Japan; htake@bukkyo-u.ac.jp; 2 Department of Epidemiology and Global Health, Umeå University, 90737 Umeå, Sweden; 3 Wakayama Seikyo Hospital, Wakayama 640-8390, Japan; wa.min-iren@maia.eonet.ne.jp; 4 Department of Community Paediatrics, South Western Sydney Local Health District, Liverpool, NSW 1871, Australia; shanti.raman@health.nsw.gov.au; 5 Division of Mental Health and Wellbeing, Warwick Medical School, University of Warwick, Coventry CV4 7AL, UK

**Keywords:** inequalities, mothers’ and children’s health and well-being, COVID-19, Japan, capability

## Abstract

Marginalised families faced significant challenges during the COVID-19 pandemic. This study explores inequalities in Japanese mothers’ and children’s health and well-being and family economic stability before and during the pandemic. Data sources were as follows: nationwide surveys in 2019 and 2021 of families with children using medical institutions across Japan; infant mortality and adolescent suicide rates between 2018 and 2021 from publicly available sources. Outcomes by poor and non-poor families were compared for 2019 and 2021 using simple descriptive statistics. Poor mothers’ part-time working increased from 41% to 61% and regular employment was reduced by two thirds. The well-being of poor mothers worsened from 39% to 55%. Employment opportunities and well-being did not change for non-poor mothers. School subsidies among poor families increased from 23% to 55%. The infant mortality rate (IMR) among unemployed families increased significantly from 12.9/1000 to 18.2/1000 between 2018 and 2021 compared with a decreasing overall IMR from 1.9/1000 to 1.7/1000. Suicide rates in 10–19-year-olds increased over the same period although no socio-economic indicators were available. Inequalities in mothers’ and children’s health and well-being indicators and family economics increased between 2019 to 2021 in Japan. This study cannot attribute causes but suggests a possible role of the pandemic.

## 1. Introduction 

Japan has enviable child health indicators, with an overall infant mortality rate of less than 2 per 1000 live births [1]. One of the characteristics of Japan is an ageing population and a decreasing proportion of children. The population rate of 0–14-year-olds is 12% of the whole population. It was the lowest in the world in 2021 [2]. Despite these health indicators, the wealth gap in Japan is increasing. Japan’s relative income gap, measured as the gap between the national median income and the bottom 10% of households with children, was 60% in 2014 [3]. It was the 10th out of 41 OECD countries in 2014. The relative child poverty rate below 50% of the median income was 15.8%. It was 26th out of 42 OECD countries in that year [4]. Public social spending on families was 2.0% of GDP in 2019, 24th out of 40 OECD countries [5]. Child poverty remains high in Japan, and public social expenditure on families is considerably lower than in other comparable countries, which has contributed to the high proportion of single mothers that rely on paid employment [6]. A 2015 report on child poverty in Japan by the Nippon Foundation [7] stated that “children raised in households in relative poverty, particularly single parent households, were at an extreme disadvantage in terms of medical care, meals, schooling, and prospects for higher education, and there is a clear trend of these children being unable to escape from poverty in the future”. The report stressed that a reduction in child poverty would improve the current living standards and future prospects of the disadvantaged as well as benefit the Japanese economy. They estimated the economic cost of child poverty stating “…the economic loss of leaving child poverty unaddressed comes to approximately 2.9 trillion yen (1.9 billion USD) for just one school year of children, and the government’s fiscal burden increases by 1.1 trillion yen (0.7 billion USD)”.

The direct effects of COVID-19 on children in Japan were not severe. One example is the death rate among positive cases, which was from 0.001 for teenagers to 0.003% for children under ten years old between September 2022 and January 2023 (Ministry of Health, Labor and Welfare) [8]. Therefore, the impact was probably less than RSV infection or influenza. In addition, Kawasaki disease-like cases and multisystem inflammatory syndrome in children (MIS-C) were rare in Japan [9]. On the other hand, the indirect effects of the pandemic have been profound for children and their families all over the world [10,11,12]. In Japan, the number of abuse consultation cases increased during the pandemic, increasing by 6% in 2020 and continuing to increase up to 2022 (Ministry of Health, Labor and Welfare) [13]. We published a mixed methods study [14] including stress scales from children experiencing social and financial disadvantage and free-text comments from them between August and November 2020. This cohort of vulnerable children in Japan had high overall stress scores during the early part of the pandemic. 

While the health of the Japanese population overall is excellent, there is evidence of significant differences in some indicators between those families living in poverty compared to the mainstream population. While COVID-19 poses the same risk of infection for everyone, the indirect effects may be greater than the direct effects for children, causing more significant difficulties for families from economically vulnerable backgrounds. 

As in Japan, globally, the COVID-19 pandemic represented a major challenge to the health and well-being of families with children, and families living in poverty and poor social circumstances were particularly vulnerable to the adverse effects of the periods of lockdown and school closures instituted by governments to control the spread of the virus [15,16]. School closures and other social distancing measures in the UK adversely affected disadvantaged, vulnerable families with children due to overcrowded living conditions, additional expenditures due to maintaining heating, providing food and the pressures of child care when schools closed [17]. The UNICEF and Save the Children joint report [18] suggests that school closures worsened children’s home environments in economically disadvantaged households globally due to deteriorated economic conditions and a lack of private educational opportunities, and the Japanese government’s internal analysis suggests a similar effect of school closures on children in economically disadvantaged households in Japan [16].

Our aims were, therefore, to describe the differences in children’s and families’ health and well-being between poor families (those living in relative poverty) and those families not in poverty in Japan over the period 2018–2021, which included the COVID-19 pandemic. We used information from national surveys of households with children conducted in 2019 and 2021, where data on household income and mothers’ and children’s health and well-being were available. We also gathered data on publicly available child health indicators, with a breakdown according to socioeconomic status where possible. 

## 2. Methods

### 2.1. Study Design

This descriptive study used the following:

- A questionnaire, designed in 2019, prior to the COVID pandemic, to provide data on the impact of poverty and socioeconomic insecurity on families in Japan. The decision to repeat the survey in 2021 was taken to explore the additional impact of the pandemic on families.
- Publicly available child health indicators from national data, wherever possible, with a breakdown according to socioeconomic status.

#### 2.1.1. Health and Well-Being of Families with Children Questionnaire

We designed questionnaires on the health and well-being of families with children that anyone could complete if they cared for children at home. The questionnaires were in four parts. The common section contained 50 questions to all respondents. It included family composition, living environment, mother’s (father’s) employment status, household income, mother’s educational background and health situation, etc. Section 1 was more than 30 questions about preschool children over three years old. It included the child’s attendance at preschool, health conditions, daily foods, vaccination, etc. Section 2 was more than 30 questions about school-age children (elementary and junior high school students) between six and fifteen. It included similar questions for preschool children. Section 3 was more than 30 questions for children between ten and fifteen. Children answered this section. It included children’s daily routines, belongings, learning situations, parental relationships, after-school activities, etc.

We designed the questionnaires so that each question group could only be finished by completing all. The first questionnaire period was from June to July 2019, and the second one was from September to October 2021.

#### 2.1.2. Sampling and Data Collection

We conducted national surveys of households with children jointly with the Japan Federation of Democratic Medical Institutions, Japan’s second-largest medical group, using convenience sampling undertaken by staff at the group’s medical institutions. Out of more than 600 medical institutions in this medical group located in all prefectures, posters and information about the questionnaire were distributed to approximately 250 medical institutions, including paediatric institutions; 30 dental medical institutions, including paediatric dentistry; and about ten pharmacies near leading paediatric clinics. After distribution, we telephoned or mailed each clinic’s director and/or paediatrician for cooperation before each year’s data collection commenced. We also requested a notice from the medical group centre appealing for cooperation in this research. During the survey period, the degree of participation and collaboration was left to the discretion of each medical institution. 

Families attending medical services of the medical group’s institutions accessed the questionnaires via a QR code displayed on posters and leaflets. Completion was by smartphone, and completed questionnaires were sent directly to the lead researcher’s email (HT). We provided incentives to 300 participants with gift vouchers in a lottery both years.

Responses from families receiving public assistance in 2021 were poor; therefore, we excluded households receiving public assistance from the 2019 questionnaire in the analysis, so as to make the populations comparable.

#### 2.1.3. Relative Poverty Measure

The relative poverty line is half the median income from the large-scale National Questionnaire of Living Conditions conducted by the Ministry of Health, Labor and Welfare in 2019 and 2022 [19,20]. This is a standard international measure of relative poverty and is not a composite measure. Households below this line we defined as “families in relative poverty” and households above this line as “non-poverty families”. Because the income question asked for a range of income, such as the JPY 3 million range [≈USD 20 thousand], households with incomes including the poverty line were excluded from statistical analysis as “borderline” each year. Respondents could choose “don’t want to answer the question” in several questions. So, not all questions were answered completely by all respondents, giving different denominators for each question.

#### 2.1.4. Selection of Outcomes for Analysis

The complete questionnaire included a total of over a hundred questions, but, for the purposes of this study, we included in the analysis only the questions that were comparable in the two periods where a difference in responses by socioeconomic status might be expected due COVID-19. The questions from which the selected outcomes were derived are listed in Table S1 in the Supplementary Material. Where more than two responses were available, outcomes were dichotomised, as shown in the table. 

### 2.2. Other Child Health Indicators

We gathered data on publicly available child health indicators from national data, wherever possible, with a breakdown according to socioeconomic status. Infant mortality rates between 2018 and 2021 were extracted from “e-Stat”, which is a portal site for Japanese Government Statistics [21]. Suicide numbers and rates of adolescents between 2018 and 2022 were extracted from the suicide statistics of the National Police Agency [22].

### 2.3. Data Analysis

The data collected for this study were descriptive, and a complex analysis designed to isolate the causal effect of the pandemic was not feasible. For this reason, we used simple descriptive statistics to analyse the quantitative data, with the Chi-square test and Mann–Whitney U test with the statistical software “EZR (easy R, ver. 1.55)”. 

## 3. Results

### 3.1. Health and Well-Being of Families with Children Questionnaire

The socio-demographic characteristics of the samples in 2019 and 2021 are shown in Table 1. The median age of mothers in 2021 was one year older than in the 2019 sample, and the proportion of single mothers was higher in 2019 compared with 2021. In all other socio-demographic characteristics, the samples were similar. 

The total responses were 1856 in 2019 and 1439 in 2021; valid responses were 1766 (94%) in 2019 and 1371 (95%) in 2021. The relatively poor and non-poor families were 156 (8.8%) and 1472 (83%) in 2019, and 115 (8.4%) and 1174 (86%) in 2021. The details of each number and percentage are shown in Table 2.

We compared data selected for relevance to the indirect effects of COVID-19 on mothers and children between 2019 and 2021 in non-poor families and families in relative poverty.

Table 3 shows that mothers’ part-time working increased from 41% to 61% (*p* = 0.074) for families in relative poverty between 2019 and 2021, and regular employment was reduced by two thirds. Part-time working among mothers in non-poor households did not change significantly. Mothers’ spending compared with fathers became more unequal in families in relative poverty, whereas mothers’ and fathers’ spending became more equal in non-poor families.

The self-reported well-being of mothers worsened from 39% to 55% significantly in families in relative poverty (*p =* 0.018) but increased in non-poor families (*p* = 0.027). The percentage of those receiving school attendance assistance increased by more than twofold in families in relative poverty in 2021 (*p* < 0.001) but did not change significantly among non-poor families. The attitude to public assistance in families in relative poverty changed: the ‘not necessary’ rate decreased by about three quarters, and that of ‘don’t want to receive’ increased by 1.6 times.

The percentage of completed cases of the influenza vaccine in school-age children was lower among children from families in relative poverty compared with those in non-poor families in 2019, and whereas the percentage increased significantly in non-poor families in 2021, the rate of completed influenza vaccination remained low in poor families. 

Two markers of deprivation among children showed differences between poor and non-poor families. The percentage of children eating breakfast alone doubled in 2021 compared to 2019 in families in relative poverty but remained the same in non-poor families. The proportion of school-aged children from families in relative poverty reporting a quiet space to do their homework decreased by more than 15% but did not change among children in non-poor families.

**Figure 1:**
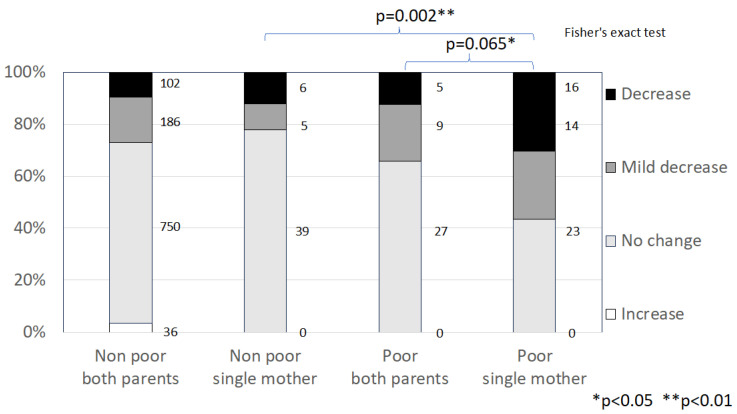
Income change reported by families in the 2021 sample.

Figure 1 shows income change during the pandemic in 2021 by poor and non-poor families, further stratified by whether they were couples or single-mother households. Poor single-mother families experienced a greater decrease in income than the other three groups, including non-poor single-mother families. 

### 3.2. Other Child Health Indicators

**Figure 2:**
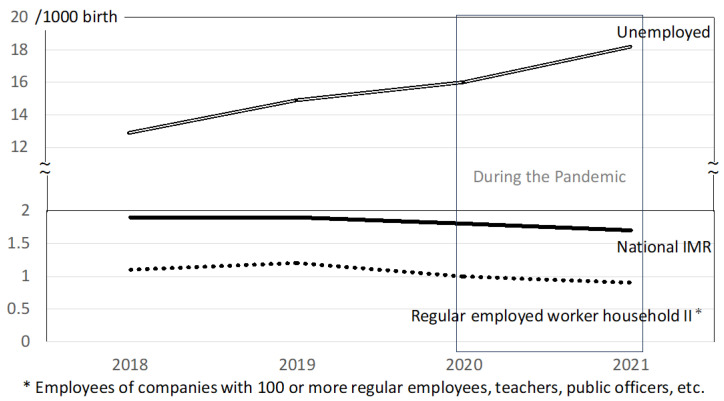
Annual trends in infant mortality rate (IMR) in Japan, by occupation of household.

Based on published Japanese government e-Stat data, Japan’s infant mortality rate (IMR) steadily decreased from 1.9/1000 live births to 1.7/1000 from 2018 to 2021 (Table 4). The IMR in households without workers increased from 14.9 to 18.2, while rates in all employment groups followed the decreasing trend in national IMR (Table 4). Figure 2 shows annual trends for four recent years. Japan’s IMR and those of households in various employment categories showed slightly decreasing trends during the pandemic. The IMR among households without workers increased to 2.0/1000 from 2018 to 2019 and 3.3/1000 during the years of the pandemic, suggesting a greater impact of the pandemic on infant health in these families compared with more advantaged families in the rest of the population.

The number of suicides in children 10–19 years old increased by more than 100 in 2020 from the previous year. For the first time, the rates are more than 2 per 100 thousand in 10–14 years old and more than 10 in 15–19 years old in 2020 (Table 5). And the high rates have been continuing in 2021 and 2022.

## 4. Discussion 

Based on questionnaires completed by mothers in 2019 and 2021 and routine data published by e-Stat annually between 2018 and 2021, we provide evidence of increasing inequalities between poor and non-poor Japanese mothers and their children across a range of outcomes. The findings indicate that poor mothers’ employment, well-being and share of the household income all decreased over the period 2019 to 2021 compared with little change for non-poor mothers. Household finances decreased significantly, particularly among poor, single-mother households, and reliance on school attendance subsidies increased among poor, but not non-poor, households. On the other hand, even though the financial situation of poor families worsened, there was a reluctance to receive public assistance. Indicators of children’s deprivation (eating breakfast alone and no quiet space to do homework) increased in poor families but did not change in non-poor families. Between 2018 and 2021, infant deaths in Japan fell from 1.9/1000 to 1.7/1000 live births but increased sharply in the poorest households with no adult in work from 12.9/1000 to 18.9/1000 in the same period.

Suicide rates increased from 5.3/100,000 in 2018 to 7.4/100,000 in 2022 among children and youth aged 10–19 years in Japan. The increasing trend over the period of the pandemic was noted in both boys and girls, and suicide was the leading cause of death in the 10–19-years age group. Although the available dataset does not include information on social circumstances, it is reasonable to assume rates will be highest among socially vulnerable children and youth. Goto and colleagues, examining both the timing of changes in suicide rates and possible explanations for changes they observed in children aged 10–19 years between 2020 and 2021, identified several factors associated with the increase in suicides including family-related concerns, mental illness, social concerns and academic concerns [23]. No other country has experienced increases, not just in adolescent suicides but suicides across a range of age and sex groups, like Japan has; therefore, continued vigilance is essential to ensure that children and young people’s mental health is supported [24].

The COVID-19 pandemic and measures taken to control its spread represented a major challenge to parents and children due to both its direct and indirect effects. Family and child health problems due to the indirect effects of the pandemic have been documented [25]; however, the literature on family and child health inequalities in the pandemic is limited. The long-term effects of the pandemic on children and families have been identified as social, emotional, behavioural, educational, mental, physical and economic and most likely to affect ethnic minorities and low-income children and families [26]. Pre-existing inequalities have been shown to contribute to the disproportionate impact of the pandemic on the health and well-being of children in marginalised groups in the UK [26] and the USA [27]. The pandemic has also disproportionately affected the mental health of children who are ethnic minorities [10]. Pre-existing inequalities and the indirect effects of the pandemic combining to exacerbate each other has been characterised as a *syndemic* [28,29]. Studies from Australia [30], the UK [31] and Spain [32] all describe child health inequalities during the years of the pandemic. Our findings suggest a significant increase in inequalities across a range of outcomes which may be attributable to the pandemic; however, as indicated above, the study design does not permit the isolation of the causal effect of the pandemic or exclude other potential changes in Japanese society over the same period that may have contributed to the increase in inequalities.

In our study of stress levels in a cohort of at-risk young people in Japan in 2020, we found that many children experienced difficulties. Many also showed positive coping, being supported by family and friends. However, a small number of children exhibited poor coping, and we advocated that a public health response to COVID-19 should be responsive to the needs and concerns voiced by CYP and be evidence-informed with respect to school closures [14].

While the increase in child and family inequalities we describe cannot be attributed solely to the pandemic, the pandemic did expose the vulnerability of poor families in Japan. Household incomes and employment of mothers were differentially adversely affected among poor families. Policies are urgently needed to optimise the well-being of children and their mothers living in vulnerable households. These should include measures to reverse the increase in the income gap [3] by increasing the incomes of the poor and increasing social spending on families above the current level of 2% [5]. These measures will contribute to reducing the child poverty rate. 

The stigma associated with the receipt of public assistance in Japan is reflected in our findings that a high proportion of respondents did not want to receive it. The Ministry of Health, Labor and Welfare has only once officially published the percentage of welfare recipients relative to the number of low-income households below welfare standards, based on a 2007 survey. According to the report, the rate of receiving households based on income was 15%, and even when assets were considered, the rate was 32% [33]. The rate of uptake in Japan is lower than in other Organisation for Economic Co-operation and Development (OECD) countries that have rates from 40 to 90% [34]. This suggests a significant role of stigma in discouraging the uptake of public assistance for children and families with low incomes. A study comparing stigma and shame and the experience of poverty in Japan and the UK [35] concludes that “stigma and shame perform important functions within both these societies as a means to legitimate the continued existence of poverty within their social systems”. Especially in Japan, people’s attitudes are influenced by the conservative policy of familialism with its origins before World War 2, which confines welfare to family issues and attempts to limit public responsibility [36]. The success of measures to reduce poverty and vulnerability will require civil society and political decision-makers to counter stigmatising attitudes.

Against this background, we must focus on optimising children’s capability [6]. In other words, we should pay serious attention to the fact that children have the right to realise what they want to be and do now and to picture what they want to become in the future. Current welfare or family policies are influenced and limited by historical legacies. However, we argue that regardless of the welfare regime, it is important to make our societies more equal through redistributing economic resources and advocating equal rights for individual children to be recognised as independent and have dignity even during the pandemic. This is aligned with the ambitions of the 2030 Agenda of the United Nations Sustainable Development Goals (2015) in leaving no child behind [37].

### 4.1. Strength and Limitations

A strength of this study is the collection of similar questionnaire data before and during the pandemic that enabled descriptive analysis of changes in family circumstances among poor and non-poor households. Although the samples in 2019 and 2021 were cross-sectional, they were similar in socio-demographic characteristics (see Table 1) and drawn from the same sampling frame, making the assumption of data comparability valid. The following limitations should be considered in interpreting the results. The data collected are descriptive and, as indicated in the methods, do not allow for causal inference. Our findings only allow us to suggest an association with the pandemic. The sampling frame for the surveys was based on a clinical population at Japan’s second-largest medical group rather than a nationally representative population. The non-representativeness of the samples limits the extent to which the findings can be applied to Japan as a whole; however, the samples included similar proportions of poor and non-poor families measured using the nationally (and internationally) recognised measure of relative poverty, allowing us to identify inequalities that are likely to have been replicated across the population. The 2021 questionnaire was not identical to the 2019 questionnaire as the families receiving public assistance were excluded. So, the data of the families receiving it in 2019 were also removed in the comparison between 2019 and 2021. The surveys were conducted nationwide except for two prefectures. The official data on suicide rates do not include the socioeconomic backgrounds of children and young people, so we were unable to analyse whether social inequalities exacerbated suicide rates.

### 4.2. Further Research

Research into the social determinants of maternal and child health in Japan is limited. The current study suggests the need for a programme of research exploring equity across a range of child health and development outcomes and the challenges of parenting for families in poverty. Specifically, the social determinants of infant, child and adolescent mortality, especially the increasing suicide rates, will be necessary to understand the drivers of mortality in Japan and inform measures to reduce mortality rates. 

## 5. Conclusions

Child health, public health policy and planning leaders in Japan should respond with some urgency to the findings of this study. Inequalities in mothers’ and children’s health and well-being indicators increased during 2018 to 2021, suggesting an association with the pandemic in Japan. Poor families experienced increased adverse outcomes, while non-poor families showed little change. There were clear correlations between families’ economic stability and children’s functioning at home and school. Suicide rates among adolescents in Japan rose dramatically in the same time frame. To optimise children’s capability through mothers’ and children’s health and well-being and increase children’s resilience, governments should do more to increase family socioeconomic stability. We would recommend that the Japanese government direct more resources proactively at poor families, given the evidence that pro-poor strategies can have important effects on coverage in underserved populations and equity in use across income groups [38].

## Figures and Tables

**Table 1:** Socio-demographic characteristics of 2019 and 2021 samples.

Socio-Demographic Factors	2019 Sample (*n* = 1766)	2021 Sample (*n* = 1371)	*p* Value
Mothers’ age (median) [25–75%]	39 [35.00, 43.00]	40 [36.00, 45.00]	*p* < 0.001 *
Household members (median) [25–75%]	4 [3.00, 5.00]	4 [3.00, 5.00]	*p* = 0.576 *
Number of children <19 years old in house hold (mean) [25–75%]	2 [1.25, 2.00]	2 [1.00, 2.00]	*p* = 0.895 *
Single mother household (%)	9.5%	8.6%	*p* < 0.050 **
Low maternal education (high school dropout or below) (%)	3.2%	2.9%	*p* = 0.779 **
Low paternal education (high school dropout or below) (%)	4.1%	4.9%	*p* = 0.357 **
House ownership: Rented (%)	24.0%	24.1%	
Repaying (%)	60.2%	61.1%	*p* = 0.742 **
Finished repaying (%)	15.8%	14.8%	
Relative poverty (%)	8.8%	8.2%	*p* = 0.166 **

*: Mann–Whitney U test. **: Chi-square test.

**Table 2:** Number and percentage of valid answers in total and per each type of economic background, Health and Well-being of Families questionnaire.

	Subject	Total	Missing *	Valid	Poverty **	Non-Poverty	Borderline Range ***
2019	Common Questions	1856	90	1766 (94%)	156 (8.8%)	1472 (83%)	138 (7.8%)
	Questions relating to pre-school children	995	51	944 (95%)	88 (9.3%)	778 (82%)	78 (8.3%)
	Questions relating to school children (6–15 y)	1228	57	1171 (95%)	100 (8.5%)	973 (83%)	98 (8.4%)
	Questions relating to 10–15-year-olds	332	14	318 (96%)	32 (10%)	250 (79%)	36 (11%)
2021	Common Questions	1439	68	1371 (95%)	115 (8.4%)	1174 (86%)	85 (6.2%)
	Questions relating to pre-school children	500	22	478 (96%)	31 (6.5%)	419 (88%)	28 (5.9%)
	Questions relating to school children (6–15 y)	772	35	737 (95%)	62 (8.4%)	633 (86%)	42 (5.7%)
	Questions relating to 10–15-year-olds	226	9	217 (96%)	16 (7.4%)	184 (85%)	17 (7.8%)

* Missing: There were no income data. ** Poverty: Relatively poor families’ disposable income below 50% of median. *** Borderline range: The income question asks about income ranges (i.e., between 300 million and 400 million yen). So, when the income range possibly includes the poverty line, their data was excluded as “borderline”.

**Table 3:** Comparison of key indicators from the Health and Well-being of Families questionnaire between 2019 and 2021 in non-poverty and poverty families.

Key Indicators	Socio-Economic Category	2019 Survey (%)	2021 Survey (%)	*p* Values Comparing Poor and Non-Poor
Mother’s part-time work	Poor	54/133 (41%)	60/98 (61%)	0.074 *
	Non-Poor	443/1278 (35%)	317/1013 (31%)	0.227
Mother’s expenses always put off	Poor	31/108 (29%)	26/56 (46%)	0.018 **
	Non-Poor	247/1316 (19%)	128/993 (13%)	<0.001 **
Mother’s self-reported well-being (<average)	Poor	54/138 (39%)	65/119 (55%)	0.018 **
	Non-Poor	437/1248 (35%)	278/915 (30%)	0.027 **
School attendance subsidy	Poor	36/156 (23%)	62/113 (55%)	<0.001 **
	Non-Poor	48/1472 (3%)	51/1173 (4%)	0.174
Public assistance (Not necessary)	Poor	82/149 (55%)	47/113 (42%)	0.042 **
	Non-Poor	1219/1425 (86%)	1006/1173 (86%)	0.919
Public assistance (Do not want to receive)	Poor	41/149 (28%)	51/113 (45%)	0.005 **
	Non-Poor	177/1475 (12%)	140/1173 (12%)	1.000
Influenza vaccine for school-age children	Poor	46/101 (46%)	31/62 (50%)	0.695
	Non-Poor	550/973 (57%)	421/633 (67%)	<0.001 **
Eating breakfast alone	Poor	12/98 (12%)	16/60 (27%)	0.037 **
	Non-Poor	127/963 (13%)	78/633 (12%)	0.971
A quiet space to do homework	Poor	77/98 (79%)	38/60 (63%)	0.057 *
	Non-Poor	844/963 (88%)	556/633 (88%)	0.971

* significant at 90% level; ** significant at 95% level.

**Table 4:** Infant mortality rate in Japan between 2018 and 2021 (data source: e-Stat from the Japanese government).

	Year	National Rate	Regular Employed Worker Household I *	Regular Employed Worker Household II **	Farm Household ***	Self-Employed Household ****	Others *****	Unemployed ******
IMR	2018	1.9	1.6	1.1	4.1	1.8	3.5	12.9
	2019	1.9	1.6	1.2	5.6	1.6	2.8	14.9
	2020	1.8	1.7	1	1.9	1.4	3.1	16
	2021	1.7	1.5	0.9	3.1	1.7	2.9	18.2

*: Households of regular employees of companies, private stores, etc. with 1 to 99 employees; **: households of regular employees and executives of companies and government offices except regular worker households; ***: households with only farming or farming and other work; ****: households running independent businesses, commerce and industry, service businesses, etc., by themselves; *****: households with other jobs that do not apply to the above; ******: households without workers; IMR: infant mortality rate.

**Table 5:** Suicide numbers and rates/100,000 in Japan in children and adolescents between 2018 and 2021.

	Year	10–19 Years	10–14 Years Old			15–19 Years Old		
			Total	Boys	Girls	Total	Boys	Girls
Suicide N (per 100,000)	2018	599 (5.3)	99 (1.9) *	66 (2.4)	33 (1.3)	503 (8.7) **	307 (10.3)	196 (6.9)
	2019	659 (5.9)	90 (1.7) *	47 (1.7)	43 (1.7)	563 (9.9) **	385 (13.2)	178 (6.4)
	2020	777 (7.0)	122 (2.3) **	64 (2.3)	58 (2.2)	641 (11.4) **	397 (13.8)	244 (8.9)
	2021	749 (6.9)	128 (2.4) **	60 (2.2)	68 (2.6)	632 (11.5) **	380 (13.4)	252 (9.4)
	2022	796 (7.4)	119 (2.3) **	62 (2.3)	57 (2.2)	633 (11.5) **	383 (13.8)	279 (10.6)

*: second reason for death in age group; **: top reason for death in age group.

## Data Availability

Data available on request due to restrictions for privacy and ethical considerations. The survey data presented in this study are available on request from the corresponding author. The survey data are not publicly available due to subject confidentiality.

## Supplementary Materials

The following supporting information can be downloaded at: https://www.mdpi.com/article/10.3390/children11030330/s1, Table S1. Questions and available responses for key indicators.
