# Supplementary material for: Did Inequalities in Mothers’ and Children’s Health and Well-Being in Japan Increase through the Pandemic? Evidence from Nationwide Surveys and Routinely Collected Data
Source: Children (Basel). 2024 Mar 9;11(3):330. doi: 10.3390/children11030330 (PMC10969302; doi:10.3390/children11030330)
Supplement: Supplementary file 1 [file children-11-00330-s001.zip › children-2869489-supplementary.pdf]

Table S1 : Questions and available responses for key indicators

Key indicators	Category	Available Responses
**Mother's part-time work**	**Part-time work**	[Part-time] [Temporary or Non-member contract]
	**Others**	[Full-time] [Self-employed] [Unemployed] [House wife] [Others]
**Mother's expenses always put off**	**Yes**	[I always put myself off] [I spend less on myself than my partner]
	**Others**	[I spend on myself in the same way as my partner]
**Mother's self-reported well-being**	**<average**	[Unhappy] [Comparatively unhappy]
	**Others**	[Happy] [Comparatively happy] [None of them]
**School attendance subsidy**	**Yes**	[We receive it]
	**Others**	[We don't receive it] [I don't know which one]
**Public assistance**	**Not necessary**	[There is no need to receive it]
	**Others**	[I don't want to receive it] [I want it but haven't consulted] [I asked about it but was refused by the welfare department]
	**Don't want to receive**	[I don't want to receive it]
	**Others**	[There is no need to receive it] [I want it but haven't consulted] [I asked about it but was refused by the welfare department]
**Influenza vaccine for school-age children**	**Yes**	[The child was vaccinated at the required frequency]
	**Others**	[The child took it but couldn't complete] [The child didn't take it] [I didn't know how the child could take it]
**Eating breakfast alone**	**Yes**	[The child eats alone]
	**Others**	[The child mostly eats with all the family] [The child eats with some of the family]
**A quiet space to do homework**	**Yes**	[Child's room and desk] [Child's desk and shared room] [Shared room and desk] [Child's desk in the living room] [Shared desk in the living room]
	**Others**	[There is no special study space]
